# Agraphia due to Conversion Disorder (Functional Neurological Symptom Disorder) in an 8-year-old boy: a case report

**DOI:** 10.3389/fpsyt.2025.1687215

**Published:** 2025-10-24

**Authors:** Nhu Minh Hang Tran, Tran Tuan Anh Le, Vu Quoc Huy Nguyen

**Affiliations:** Hue University of Medicine and Pharmacy, Hue University, Hue, Vietnam

**Keywords:** conversion disorder, functional neurological symptom disorder, agraphia, psychological defense mechanisms, secondary gains

## Abstract

Conversion disorder, a psychiatric condition characterized by neurological symptoms without organic etiology, is rare in children but can significantly impair functioning. This case report describes an 8-year-old boy presenting with sudden onset of hand tremors, inability to write, and recurrent abdominal pain following a three-month summer vacation and increased academic demands. Despite extensive neurological evaluations (MRI, EEG, EMG) showing no organic abnormalities, symptoms persisted, exacerbated by social isolation and secondary gains from family and school accommodations. Treatment with play therapy, cognitive therapy emphasizing fairness, and parental guidance to reduce overindulgence led to rapid symptom improvement within days. Follow-up over twenty months demonstrated sustained recovery and successful school reintegration. This case highlights the interplay of academic stress, psychological defense mechanisms, and secondary gains in pediatric conversion disorder, underscoring the efficacy of tailored psychological interventions and the importance of addressing familial dynamics to mitigate symptom persistence.

## 1 Introduction

Conversion disorder, also known as functional neurological symptom disorder, manifests as neurological or somatic symptoms without identifiable organic pathology, often triggered by psychological stressors (1, 2). In children, it is less common but can present as motor such as tremor, jerks, or dystonic movements; and gait abnormalities, sensory such as absent skin sensation, vision, or hearing, or functional impairments, such as agraphia (loss of writing ability), reflecting underlying emotional or environmental stressors (3, 4). Psychological defense mechanisms, such as regression or conversion, and secondary gains, such as increased attention or avoidance of responsibilities, may sustain symptoms, complicating diagnosis and management (5, 6). Diagnosing conversion disorder in children is challenging, requiring a multidisciplinary approach, thorough clinical evaluation, and extensive testing to rule out organic causes, a process that can be time-consuming and costly (7). Protective mechanisms, including psychological defenses and supportive environmental factors, play a critical role in both symptom manifestation and recovery. This case report describes a rare presentation of conversion disorder in an 8-year-old boy with acute agraphia, highlighting the influence of environmental, socio-cultural, and psychological factors, as well as the diagnostic and therapeutic challenges involved.

## 2 Case presentation

### 2.1 The context of situation

An 8-year-old boy, the second son in a family with two brothers (his older brother is 11 years older), whose mother is a farmer and father is a goldsmith, presented with significant hand tremors and inability to write. The patient had completed Grade 1 with excellent academic results. Prior to onset these symptoms, his school-related skills including reading, writing, and mathematics etc. … were fully age-appropriate and without any difficulties. The symptoms first appeared in June, 2022, After nearly two-month summer vacation filled with play, he began additional tutoring to prepare for second grade. During this period, his teacher and family observed frequent hand tremors and slight jerking movements when writing, leading to progressively deteriorating handwriting and eventual inability to write with his right hand.

Upon returning to school, the child was trained to write with his left hand, which he managed temporarily. Concurrently, his mother reported recurrent episodes of abdominal pain lasting several hours, occurring at any time, causing intense crying and occasional fainting, with elevated blood pressure and pulse. Despite extensive hospital evaluations, no organic cause was identified, and the pain typically resolved within one day of admission without specific treatment. Subsequently, tremors developed in the left hand, resulting in an inability to write with that hand as well. The school accommodated this by exempting him from written assignments, allowing oral responses, and arranging for a peer to transcribe his dictated answers during examinations for subjects including Vietnamese, mathematics, and social-nature. He achieved outstanding grades in both semesters of second grade using this method. In June 2023, after completing his summer vacation following Grade 2, the boy continued to experience writing difficulties. His mother sought medical evaluation in preparation for his transition into Grade 3. During this period, he was admitted to several general hospitals, where he underwent investigations including brain and spinal MRI, electromyography (EMG), and electroencephalography (EEG), all of which revealed no abnormalities. Similar investigations conducted in the previous year had also shown normal results. By the end of June 2023, he was referred to a child psychiatrist for further assessment and treatment.

### 2.2 Clinical findings

Clinical evaluation revealed a lively, agile child with prompt responses to questions and a prompt attachment to his mother.

Physical examination showed normal muscle strength (5/5) and deep tendon reflexes. When asked to draw a favorite picture under observation, he exhibited significant tremors and struggled, but completed a car drawing when unobserved. Apart from the inability to write and occasional tremors during eating, fine motor examination revealed preserved function in other tasks. The child was observed holding a mobile phone securely, catching and throwing a ball effectively, and using scissors to cut out complex shapes such as circles and cars from coloring books.

During the “three wishes” technique, he expressed desires to excel academically, for his parents’ health, and for better treatment from classmates.

During the clinical interview, the boy appeared strongly attached to his mother. He resisted her leaving the room and, unless actively engaged in activities, often preferred to hold her hand. He also reported feeling anxious whenever his mother left the house. When asked to elaborate on his worries, the boy simply stated, “I don’t like my mother going out; I want her to stay with me.” He did not express fears of being abandoned, nor did he report nightmares or separation-related distress. According to his mother, at home he liked to stay close to her and frequently shared stories about his school experiences with her. Interviews also revealed the child as affectionate and generous, frequently sharing learning materials and snacks with friends, and highly responsive to praise and recognition, becoming tearful and sad when these needs were unmet. The mother also reported that the child sometimes exhibited tremors when eating, spilling food and requiring both hands to stabilize a spoon, though these were less frequent than writing-related tremors. Other fine motor skills remained intact. The boy shared that socially, he rarely interacted with peers, describing them as “unkind and selfish,” and preferred playing with older or younger children, spending recess coloring or drawing. When asked why he perceived his classmates as unkind and selfish, the boy gave concrete examples. He reported that when peers forgot their learning materials or absent in the class, he often lent them his pens or notebooks. However, when he occasionally needed colored pencils or pen, his classmates refused to share. He also noticed that they only interacted with him when they needed academic help or to borrow his school supplies. His hobbies included physical activities (e.g., shuttlecock kicking, soccer) and coloring.

The Spence Children’s Anxiety Scale (SCAS) was administered. The child responded ‘sometimes’ to items 1, 3,12, 18, and 33; often’ to items 5, 8, 31 and 43, and ‘always’ to items 17, 26, and 38; ‘while choosing ‘never’ for all other items. These responses did not indicate clinically significant anxiety. A structured clinical interview based on DSM-5 criteria also confirmed the absence of comorbid anxiety disorders.

Neurological evaluations, including brain and spinal MRI, EEG, EMG revealed no organic abnormalities (at general hospitals).

The boy had no history of epilepsy, traumatic brain injury, or other organic medical conditions. According to the mother’s report, the boy’s developmental milestones were achieved within normal limits, with no evidence of delay in motor, language, or social domains.

His family history was also negative for psychiatric disorders, medical diseases and neuropathologies.

Before being referred to a psychiatrist, the patient had been hospitalized in the pediatric departments of several general hospitals. At these facilities, he underwent physical rehabilitation, writing practice, and acupuncture; however, his condition showed little improvement. Detailed medical records from these previous interventions were not fully available, as they were incompletely provided by the family and the prior institutions.

### 2.3 Diagnostic assessment

The boy’s presentation was most consistent with Conversion Disorder (Functional Neurological Symptom Disorder) according to DSM-5 criteria (1). He developed motor symptoms (hand tremors and inability to write), that were incompatible with recognized neurological disease, confirmed by normal MRI, EEG, EMG and clinical findings. The symptoms followed a psychosocial stressor (academic pressure and perceived unfairness among peers) caused significant functional impairment in schooling, and were not better explained by another mental disorder. Symptom resolution after psychotherapy further supported the diagnosis. The absence of alternative neurological or medical explanations aligns with diagnostic criteria for conversion disorder (1, 7), emphasizing the need for comprehensive evaluation to rule out organic causes (7). The child’s history of medically unexplained symptoms, including tremors during writing and eating and recurrent abdominal pain, is consistent with the somatization patterns seen in conversion disorder (9, 10). These symptoms likely served as an unconscious expression of unresolved emotional stress, possibly linked to earlier experiences, supporting psychodynamic theories of conversion as a defense mechanism (10–12). The child’s intact cognitive function and alertness further differentiate conversion disorder from neurodevelopmental disorders, reinforcing the psychological etiology of his symptoms. Symptom resolution after psychotherapy further supported the diagnosis.

With the clinical presentation of this boy, we also considered several alternative diagnoses. First, Separation Anxiety Disorder was considered because of his strong attachment to his mother and his discomfort when she left the room. However, this diagnosis was excluded because he did not report nightmares of separation, fears of being abandoned, or persistent worries about harm befalling his mother (1); instead, he simply expressed that he felt happier and warmer when she was present, which is developmentally appropriate at the age of eight. The Spence Children’s Anxiety Scale (SCAS) and a structured DSM-5 interview also confirmed the absence of clinically significant anxiety symptoms. Second, Factitious Disorder was considered, as the child presented with symptoms without an identifiable medical cause. This was excluded because there was no evidence of intentional symptom production, no unusual illness-seeking behaviors, and the clinical course was inconsistent with factitious disorder (1). The literature also shows that factitious disorder is usually difficult to treat, whereas in this case the boy improved rapidly and sustained recovery with psychological therapy, further supporting exclusion. Third, Malingering was considered, but excluded because there were no external incentives such as financial compensation, avoidance of responsibilities, or tangible rewards (1). On the contrary, his symptoms caused significant disadvantages in school performance and social functioning, and his cooperative engagement and long-term improvement were incompatible with deliberate symptom fabrication. In summary, the clinical presentation, developmental context, and exclusion of organic, anxiety-related, and intentionally feigned disorders all support the final diagnosis of Conversion Disorder (Functional Neurological Symptom Disorder) (1).

### 2.4 Therapeutic intervention

At psychiatric department, the child was diagnosed with conversion disorder and treated with play therapy involving drawing, coloring, and preferred physical activities (e.g., football, shuttlecock kicking). The boy received daily one-hour therapy sessions during his one-week inpatient admission. Play therapy was employed to facilitate emotional expression and provide a safe context for exploring interpersonal themes such as fairness and cooperation. Sports activities, including ball games, shuttlecock kicking were introduced to strengthen gross motor coordination and build teamwork and self-confidence. Drawing and coloring tasks were used to train fine motor skills in a relaxed, non-threatening way, indirectly supporting recovery of handwriting. Cognitive-behavioral techniques included guided reflection on experiences of fairness(e.g., discussing whether peer assistance in exams was fair), cognitive restructuring of distorted beliefs (e.g., perceiving the friends as unkind and selfish people), reinforcing positive traits, such as generosity, empathy, academic excellence, and care for his parents; and role-playing to practice alternative responses. Parental guidance was also provided, addressing the mother’s overprotection, avoiding overindulgence or fostering dependency, and minimizing secondary gains, such as special privileges from family and school for his writing difficulties and encouraging consistent behavioral reinforcement at home. The therapist also normalized the child’s experiences, acknowledging that extensive writing can cause fatigue and tremors in children, and recommended short breaks (5–7 minutes) for rest or play to improve writing endurance. Additionally, the child was guided in emotion regulation techniques and skills to identify and express emotions appropriately, which supported his ability to cope with stress and reduced symptom expression (8). This multimodal approach was developmentally appropriate, engaging, and contributed to rapid clinical improvement.

### 2.5 Follow up and outcome

Although the patient was hospitalized for only one week before discharge, structured follow-up was arranged. During the first month, he was monitored once weekly through reassessment based on maternal and child reports via video calls. In the second month, follow-up occurred once every two weeks, and from the third month onward, assessments were conducted monthly. During each follow-up, any concerns raised by the child or his mother were addressed by the therapist. From the twelfth month onward, follow-up visits were reduced to once every three months. Follow-up over 20 months, the boy showed continued symptom improvement, with the child successfully reintegrating into school (**Table 1**).

**Table 1:** Timeline table for the case presentation.

Time point	Event/clinical details	Intervention	Outcome/notes
June, 2022	First onset of symptoms: hand tremors and inability to write, soon after completing Grade 1 with excellent academic results and normal learning skills (reading, writing, mathematics).	No treatment at this stage, EEG performed	Initial symptom persistence, EEG result normal
June, 2022 – May 2023	Studying at grade 2 with help from friends to writing tasks in the school, writing difficulties persisted His brother went to the university in another province	exempting from written assignments, allowing oral responses	Completing Grade 2 with excellent academic results
June 2023 (after Grade 2 summer vacation)	Writing difficulties persisted, mother sought care to prepare for Grade 3.	Evaluated at several general hospitals	Physical rehabilitation, writing exercises, acupuncture; minimal improvement
June 2023 (before Grade 3)	Hospital admissions for investigations.	Brain and spinal MRI, EMG,	All results normal; no organic pathology
End of June 2023	Referred to child psychiatrist for specialized care.	Comprehensive psychiatric evaluation including DSM-5 interview and Spence Children’s Anxiety Scale (SCAS)	No comorbid anxiety disorder identified; strong maternal attachment and peer-related concerns noted
July 2023 (first admission to psychiatry)	Psychiatric inpatient stay (1 week).	Play therapy, CBT focusing on fairness, parental guidance	Marked improvement within 2 days; discharged after 1 week
July–August 2023 (first 2 months follow-up)	Post-discharge monitoring.	Video calls once weekly in Month 1; biweekly in Month 2	Stable improvement; no relapse
September 2023 – June 2024 (Months 3–12)	Continued follow-up.	Monthly monitoring and therapist guidance	Patient returned to school and resumed normal activities
July 2024 – February 2025 (Months 12–20+)	Long-term monitoring.	Follow-up every 3 months	Sustained recovery; stable academic and social functioning

## 3 Discussion

In this discussion, we aim to analyze in greater depth the psychosocial factors contributing to the onset of the disorder in this child, as well as to review the therapeutic approach, strengths, and limitations of this clinical case. The first psychological factor contribute to the disorder would be academic pressure. The symptom onset following a relaxed summer vacation and the transition to increased academic demands in second grade, with greater writing requirements, suggests academic pressure as a key precipitating factor. This aligns with research indicating that transitions to high-demand environments can trigger conversion symptoms in children, particularly in cultures valuing academic excellence (3). The second was social isolation, evidenced by limited peer interactions and perceptions of classmates as “unkind and selfish,” likely exacerbated psychological stress. Social exclusion is a recognized risk factor for functional disorders in children, as it amplifies emotional distress (4). This case suggests that interventions fostering peer relationships could mitigate symptom severity, highlighting the importance of social skills training in treatment plans.

The child’s emotional sensitivity, generosity, and strong attachment to his mother, coupled with her indulgent parenting style, played a significant role in symptom maintenance. Excessive maternal attention and prolonged medical evaluations across multiple hospitals likely heightened the child’s anxiety and disrupted his sense of stability (13–18). This iatrogenic effect - where medical interventions inadvertently worsen symptoms - underscores the need for early psychological intervention to avoid unnecessary testing (7). Parental guidance, as implemented here, was critical in addressing these dynamics and promoting healthier family interactions.

As the youngest child with an 11-year age gap to his older brother, the boy likely received heightened pampering and protection, fostering greater dependency and reducing his ability to cope independently, particularly amid peer isolation. It is possible that the boy’s strong attachment to his mother was partly shaped by familial circumstances. For example, when he was unable to write, his mother immediately considered purchasing technological tools such as laptop, tablet, smartphone to replace handwriting, reflecting a highly protective parenting style. The perspectives of the boy and his mother provide important insights into the psychosocial dynamics surrounding the symptom presentation. The boy’s strong preference for his mother’s presence, although developmentally common at his age, appeared intensified by maternal overprotection. Literature has shown that excessive parental attention and prolonged medical consultations across multiple hospitals may inadvertently heighten a child’s anxiety and reinforce functional symptoms (13–18). In this case, the mother’s tendency to consider technological alternatives to handwriting may have further validated the symptom and disrupted opportunities for natural recovery. These observations are consistent with prior reports that parental overinvolvement can contribute to symptom persistence in pediatric conversion disorder (13–16). Furthermore, when the older brother left home for schooling, the mother may have experienced a sense of emptiness, which could have intensified her emotional investment in the younger child. These dynamics likely reinforced the child’s dependence on his mother and contributed to his psychosocial vulnerability. This dynamic intensified psychological pressure, as the undivided parental affection, especially from the mother, may have amplified his emotional reliance (19).

The coincidence of symptom onset with the older brother’s departure for university in another province suggests an additional layer of internal conflict, potentially converting feelings of separation and loneliness into somatic symptoms, compounding academic stress. In Vietnamese culture, the proverb “Giàu con út, khó con út” (wealth benefits the youngest, hardship burdens the youngest) illustrates the deep parental attachment to the youngest child, often leading to overprotection and dependency, influencing personality formation through familial environment (20, 21). This saying reflects how the youngest child inherits family fortunes or hardships, fostering reliance on parents and potentially hindering adaptive skills. In this case, such overprotection may have limited the child’s problem-solving abilities, making him more susceptible to conversion disorder and anxiety under stress, as cultural norms emphasize sheltering children while expecting filial piety and obedience (22–25).

Secondary gains, including exemptions from writing, peer assistance during exams, and heightened maternal attention, likely reinforced the child’s symptoms by providing unconscious rewards, such as avoidance of academic pressure (5). The mother’s belief in advanced modern treatments and her suggestion of technological aids (e.g., laptops or tablets for typing) further amplified these gains, as the child internalized these ideas, viewing gadgets as desirable perks and reinforcing the notion that only innovative methods could resolve his condition. This suggestion effect prolonged symptoms by diminishing confidence in traditional recovery processes and increasing dependency. The therapeutic approach, which emphasized fairness, positive traits, and disruption of these gains, proved effective, with symptom improvement within days. This rapid response highlights the efficacy of cognitive-behavioral and play therapy in addressing pediatric conversion disorder, particularly when tailored to developmental stages (26, 27).

At age 8, the child’s developmental stage, characterized by emerging moral reasoning and a need for recognition (28–30), influenced his symptom presentation. According Piaget’s developmental theory of moral judgment and other authors, which highlights children’s sensitivity to fairness around age 8 (28–31). In this case, the boy perceived his peers as selfish and unfair, noting that although he often shared his writing tools or food, his classmates rarely reciprocated. Another situation was directly related to his own academic context, while he expected fairness from others, he also allowed another child to write his exam for him, which raised the question of whether he himself was being fair to his peers. Highlighting this discrepancy helped the boy reflect on fairness not only as a right but also as a responsibility. The therapeutic success achieved through the cognitive axis of ‘justice’ is best understood by linking it to foundational theories of moral development. An 8-year-old typically functions within Kohlberg’s Preconventional Morality, specifically Stage 2: Individualism and Exchange (or *instrumental relativist orientation*) (32). In this stage, fairness is perceived as a transactional, ‘tit-for-tat’ exchange: ‘I help you, so you must help me’. The patient’s initial emotional trigger, feeling that peers were ‘unkind and selfish’ by not sharing was a perceived violation of this transactional justice.

The intervention’s breakthrough occurred when we leveraged this framework, but reframed the ethical focus from the patient as the victim of injustice to the patient as the perpetrator of an injustice. The symptom’s secondary gain had led to a significant burden: a classmate was obliged to write for him during exams. We emphasized that while his hand could not write, his illness was unfairly forcing a peer to sacrifice their own time and academic focus - a severe moral breach in his transactional worldview. Challenging the patient to view his continued symptom as an act of injustice toward a peer led to a rapid cognitive and moral shift. This reframing successfully undercut the secondary gain, aligning his recovery with a developmentally appropriate push toward a more autonomous and principled understanding of fairness (28) (Piaget’s transition from heteronomous to autonomous morality), which ultimately drove the functional recovery.

This insight provided motivation to resolve his internal conflict, reduce secondary gains, and contributed to the rapid improvement of his symptoms. Regression (e.g., spilling food, resembling infantile behavior) and conversion (translating academic stress into physical symptoms) served as coping mechanisms for overwhelming demands. These findings support psychodynamic models where unconscious processes manifest as somatic symptoms to manage stress. The focus on fairness and positive reinforcement in therapy aligned with the child’s developmental priorities, facilitating recovery.

As part of the therapeutic program, the boy engaged in daily sessions that incorporated multiple activities tailored to his developmental stage. Play therapy was used to provide a safe medium for emotional expression and to explore interpersonal themes such as fairness and cooperation (33). Sports activities, including ball games, were introduced to strengthen gross motor coordination and to foster teamwork and self-confidence (34). Drawing and coloring tasks were integrated to train fine motor control in a natural, low-pressure context, gradually rebuilding his writing-related skills (35–37). Together, these interventions promoted both emotional regulation and functional recovery, while maintaining the child’s active participation and enjoyment in therapy.

Several international case reports and cohort studies confirm similar presentations and management strategies in pediatric conversion disorder. For instance, Ozsungur et al. (2012) described a 10-year-old boy with severe conversion disorder who improved markedly with play therapy, individual psychotherapy and family involvement (38). Li et al. (2021) analyzed 66 cases in Western China, finding that parental cooperation predicted better prognosis, while comorbid anxiety or depression correlated with poorer outcomes (39). Santric et al. (2016) reported three cases initially misdiagnosed in pediatric emergency settings, later clarified as conversion disorder after psychiatric evaluation (40). Zhang Y. et al. (2023) presented a case of postoperative conversion aphonia in a child, highlighting the role of stress and rapid psychiatric intervention (41). These reports emphasize that psychosocial stressors, thorough exclusion of organic causes, and early psychological treatment are critical in management—features also observed in our case. At the same time, our therapeutic focus on fairness represents an innovative and developmentally tailored contribution not previously emphasized in the literature.

The favorable prognosis in this case may be explained by several protective factors: the boy’s young age, absence of psychiatric comorbidity, supportive family environment, and high adherence to therapy. Sustained monitoring over 20 months further reinforced recovery. Nevertheless, clinicians should remain vigilant for potential relapse, especially during periods of academic transition such as examinations or progression to higher grades, which may re-activate stress and functional symptoms.

This case has several strengths. First, it provides a detailed longitudinal account of conversion disorder in childhood, including a structured 20-month follow-up that demonstrated not only rapid improvement but also sustained recovery. Second, the therapeutic approach incorporated a developmentally tailored focus on fairness, which is rarely emphasized in the literature yet highly relevant to an eight-year-old’s psychosocial and cognitive development. Third, the diagnostic process was rigorous, involving neurological investigations (MRI, EEG, EMG), structured psychiatric assessment with DSM-5 criteria, and the use of the Spence Children’s Anxiety Scale, thereby ensuring diagnostic validity and excluding comorbid conditions and intentional symptom production.

Nevertheless, this case also has limitations and potential biases. First, cultural factors must be acknowledged, including mother’s overprotection and the Vietnamese proverb emphasizing maternal devotion, which may have contributed to the child’s dependence and influenced our therapeutic framing. Second, the hospital transfer itself and family expectations may have played a role in the rapid symptom resolution, raising the possibility of nonspecific contextual effects. Third, incomplete access to medical records from prior hospitalizations limited the comprehensiveness of the case history. These limitations suggest caution in generalizing the findings while still underscoring their educational value.

## 4 Practical implication

A key clinical implication of this report is the critical need for early recognition and culturally sensitive intervention. The patient’s initial, prolonged journey through extensive and expensive neurological evaluations (MRI, EEG, EMG) underscores a diagnostic delay that is common but avoidable with a high index of suspicion. For child and adolescent psychiatry, this case highlights the necessity of integrating the patient’s and family’s core narratives into the diagnostic formulation.

The primary stressors were rooted in the Vietnamese socio-cultural context of intense academic pressure, which led to the patient’s emotional crisis and sense of social injustice (feeling betrayed by peers). The maintenance of the symptom was cemented by the secondary gain derived from family and school accommodations (e.g., being exempted from writing homework and tests). Our case provides a practical example of how a multidisciplinary, culturally informed approach, one that understands the local academic environment and familial dynamics is essential for achieving complete and sustained remission.

In summary, this case highlights several important lessons. For clinicians, it emphasizes the importance of ruling out organic pathology while also addressing psychosocial and developmental factors. For psychiatrists and psychologists, it demonstrates the usefulness of integrating age-appropriate cognitive themes, such as fairness, into therapy. For pediatricians, it illustrates how repeated medical procedures without clear findings may exacerbate anxiety and delay psychiatric referral. For teachers and educators, it provides a reminder that functional symptoms may mask underlying psychosocial stress, and that supportive collaboration with families and clinicians is essential.

This case also highlights the complex interplay of psychological, familial, and environmental factors in pediatric conversion disorder. It advocates for holistic interventions that address underlying stressors, disrupt secondary gains, and involve family dynamics to optimize outcomes. The child’s sustained improvement and successful school reintegration underscore the potential for recovery with targeted psychological support. Together, these lessons may help professionals in recognizing, differentiating, and managing pediatric conversion disorder more effectively.

## 5 Conclusion

The hallmark of this case report lies in its description of an extremely rare symptom of conversion disorder: agraphia (inability to write) in an 8-year-old school-aged child. While pediatric conversion disorder commonly presents with gross motor or sensory symptoms (e.g., gait disturbance, pseudoseizures), agraphia is a subtle, specific higher-order cognitive dysfunction predominantly reported in adults with focal brain lesions. The presentation of agraphia in our patient manifesting as a tremor in the dominant hand that quickly extended to the non-dominant hand, yet sparing all other fine motor skills (e.g., drawing, using scissors, buttoning) strongly supports its non-organic etiology. This case is a crucial addition to the literature, illustrating that psychological distress can be somatized through highly specialized cortical functions like writing, particularly in the context of significant academic stress. Multidisciplinary evaluation was essential to rule out organic causes, while play and cognitive therapies, combined with parental guidance, effectively reduced symptoms by addressing psychological defenses and secondary gains. The case emphasizes the importance of early psychological intervention, balanced parenting, and school-based support to prevent symptom persistence. Further research into cultural and educational influences on pediatric conversion disorder could inform preventive strategies and improve outcomes.

## 6 Patient’s and mother’s perspective

The boy shared that he felt more comfortable and secure when his mother was with him. He explained that he did not like it when his mother left the room, saying simply: *“*I don’t like it when Mom goes out; I want her to stay with me.” He did not describe fears of abandonment or nightmares related to separation, but rather emphasized the warmth and reassurance he experienced in his mother’s presence. The mother reported that at home the boy often stayed by her side, holding her hand when possible, and regularly told her stories about school. She also explained that when the boy struggled with handwriting, she considered buying technological devices so that he would not have to rely on writing by hand. This perspective highlights the child’s reliance on maternal presence and the mother’s protective attitude during the illness course. The mother initially expressed hope for advanced modern treatments to fully cure her son’s condition, while harboring doubts that the symptoms might never resolve completely. She considered alternative solutions, such as purchasing a laptop or tablet to enable the child to type instead of writing by hand. The child, influenced by his mother’s views, echoed these sentiments, which inadvertently amplified secondary gains—such as acquiring appealing technological devices and avoiding handwriting tasks. This shared perspective also instilled a belief that only cutting-edge methods could heal the illness, potentially prolonging and intensifying the symptoms through suggestion and reinforced dependency. However, after 1–2 days of treatment in the mental health department with psychological therapy, the child showed symptom improvement, and the mother cooperated closely with the therapist. She developed confidence that her son could recover without advanced modern methods, by adopting appropriate interactions, providing timely support, and fostering problem-solving skills, independence, and responsibility in the child.

## Data Availability

The raw data supporting the conclusions of this article will be made available by the authors, without undue reservation.
